# *Beilschmiedia turbinata*: A Newly Recognized but Dying Species of Lauraceae from Tropical Asia Based on Morphological and Molecular Data

**DOI:** 10.1371/journal.pone.0067636

**Published:** 2013-06-28

**Authors:** Bing Liu, Yong Yang, Lei Xie, Gang Zeng, Keping Ma

**Affiliations:** 1 State Key Laboratory of Vegetation and Environmental Change, Institute of Botany, Chinese Academy of Sciences, Beijing, China; 2 State Key Laboratory of Systematic and Evolutionary Botany, Institute of Botany, Chinese Academy of Sciences, Beijing, China; 3 Laboratory of Systematic Evolution and Biogeography of Woody Plants, College of Biological Sciences and Biotechnology, Beijing Forestry University, Beijing, China; 4 University of Chinese Academy of Sciences, Beijing, China; Wuhan Botanical Garden, Chinese Academy of Sciences, China

## Abstract

China took great efforts to reforestation, even turned the long-term forest loss into a net gain, but this cannot hide the loss of species diversity due to destruction of primary forests, habitat loss, invasion of alien species, and over exploitation. Here we provide such a case by recording a dying tree species of Lauraceae from the evergreen forests of SE Yunnan of China and adjoining Vietnam. We made field collections and observations for four consecutive years from 2009 to 2012. Phylogenetic analyses were conducted based on a combined dataset from nrITS and plastid *trnL-trnF* region, *rpl16* intron, and *psbA-trnH* spacer. The results indicate that the Asiatic *Beilschmiedia* and *Syndiclis* are reciprocally monophyletic with *Endiandra* as a sister group, and both morphology and molecular phylogeny clearly suggest that the new species belongs to *Beilschmiedia*. Thus *Beilschmiedia turbinata* Bing Liu et Y. Yang is illustrated and described as new to science, color plates, line drawings, distribution map and comparison with related species are provided. This new species is similar to *B. yunnanensis* in the small and ferruginous-brown tomentose terminal buds, elliptic to oblong-lanceolate and alternate or subopposite leaves bearing the fine veinlet reticulation, but differs from the latter by the smaller flowers, the eglandular stamens of the third whorl, and the large turbinate furfuraceous fruits.

## Introduction

Global biodiversity loss is a serious problem due to habitat loss, overexploitation, biological invasion, pollution, and global climate change according to the Millennium Ecosystem Assessment [1], and it is the case in China as well. Though China took great efforts in reforestation, and even turned the long-term forest loss into a net gain, the increased forest cover mainly consists of non-native tree crops but not regenerating natural forest [2]. The primary forest was continuously cut down and transformed into monoculture plantations of non-native economic trees [2]–[4]. After privatization of former collective forests since 2008, smallholders often cut natural forests for immediate income, then plant monoculture tree crops for long-term investment [2]. Because loss of native species diversity is masked under the increasing reforestation, it is more serious than what people thought [2], [5], especially in areas with high species richness, e.g. Yunnan of China.

Southeast Yunnan of China, adjoining to Vietnam and Laos, preserves the northernmost tropical rainforests in Asia, and harbors extremely high diversity of plant species [6], with more than 7000 native species of flowering plants [7], and falls within one of the biodiversity hotspots in the world [8], but experienced a fast loss of species due to farming, logging, habitat loss, and vegetation destruction. Many species were thus eliminated from the earth before they were recognized and described. Here we describe a critically threatened tree species of Lauraceae from the evergreen forest of SE Yunnan of China based on field investigations in SE Yunnan and N Vietnam in the last four years and a detailed comparative study including morphology, anatomy, and molecular systematics analyses.

The Lauraceae are typical elements of SE Asia, and are also well represented in SE Yunnan of China [9]–[10]. The pantropical genus *Beilschmiedia* Nees is one of the largest genera within the family, containing ca. 250 species worldwide [11]–[13], and includes common trees in tropical Asia [9]. In addition to old publications of Liou [14] and Kostermans [15], a few regional revisions on this genus have recently been completed, e.g. America [16], Borneo [17], Madagascar [13], and China [18].

It is difficult to distinguish *Beilschmiedia* from its close relatives due to overlapping characters [19]–[20], e.g. *Potameia* (Madagascar), *Endiandra* (mostly in Australia, three species in China), *Syndiclis* (mostly in China), *Sinopora* (Hong Kong), and *Yasunia* (S America), which together belong to the *Beilschmiedia* group [20]. The genus *Beilschmiedia* has been recognized based on a combination of characters: cymose-paniculate inflorescences with the lateral flowers of the cymes not strictly opposite but somewhat alternate [21], trimerous flowers bearing usually nine but sometimes six fertile stamens, anthers 2-celled, and small tepals rarely persistent at the base of fruits. None of these characters, however, can distinguish this genus from its close relatives [13], [20]. The minor venation pattern is a useful character to delimit *Beilschmiedia* species, and could be divided into two groups: fine type and coarse type [16]. Cuticular characters were sometimes used to determine generic ascription [19]–[20], [22], and indeed, leaf cuticular morphology is a good marker to define *Syndiclis* [22]. We tried several times to extract cuticles of our new species, but failed, because the epidermis of this new species is strongly thickened, in which it differs from all other known Asiatic species of the *Beilschmiedia* group [22].

Molecular phylogenetics based on DNA sequencing nowadays becomes a regular approach in plant systematics. In addition to the big phylogeny of the Lauraceae [23]–[24], a few molecular systematic studies were conducted on Lauraceae to discuss the generic relationships, e.g. *Litsea* complex [25]–[27], *Neolitsea* [26]–[28], *Neocinnamomum* [29]–[30], *Actinodaphne* [26]–[27], [31], and *Persea* group [32]–[33], but the *Beilschmiedia* group was poorly sampled. For the *Beilschmiedia* group in the Tropical East Asia (*Beilschmiedia*, *Sinopora*, *Syndiclis*, and *Endiandra*), it is difficult to separate *Endiandra* and *Syndiclis* from *Beilschmiedia*. This group is poorly represented in previous molecular phylogenetic studies of Lauraceae [23]–[24], [29]. To determine the generic ascription of our new species, we sampled twelve species of Asiatic *Beilschmiedia*, two *Endiandra*, and four *Syndiclis*, which is the most extensive sampling of the *Beilschmiedia* group thus far, no DNA analysis has been applied in Lauraceae to determine the taxonomic ascription of newly described species thus far. Both nuclear ribosomal ITS and chloroplast DNA fragments (*trnL-trnF*, *rpl16* and *psbA-trnH*) were sequenced. This phylogenetic study is helpful to determine the taxonomic position of the newly recorded species in addition to morphological evidence.

## Materials and Methods

### Ethics Statement

The field investigations were carried out in collective forests in Sumawan of China and Thanh Thuy of Vietnam, which are owned by the local village, but not protected area of state-owned land. The village head of Sumawan, Mr. Maorong Tian, gave us permissions to conduct the investigations and recommended two villagers as guides for us. Đào Thiêu assisted us collecting in Thanh Thuy of Vietnam.

### Morphology and Anatomy

We surveyed three sites which cover the whole known distribution area of the new species. The voucher specimens, FAA-pickled materials, measurements, and photographs of vegetative and reproductive characters were obtained in the field. Anatomical observations of leaflet venation pattern and floral structure were taken under light microscope (Zeiss Axio Imager A1) in LSEB (State Key Laboratory of Systematic and Evolutionary Botany), IBCAS (Institute of Botany, the Chinese Academy of Sciences). *Beilschmiedia yunnanensis* Hu is close to the new species in morphology, and the two species were compared in detail.

### Assessment of Conservation Status

We estimated the population size in the field, investigated the threat factors, and assessed the endangered category according to IUCN red list criterion [34]. The distribution map and calculation of EOO (extent of occurrence) were made by usingArgGIS ver. 9.3 [35].

### Molecular Systematics

To determine the systematic position of our new species, thirty-two samples belonging to nineteen species of four genera (*Beilschmiedia*, *Endiandra*, *Syndiclis*, and *Cryptocarya*) were included in this study. All but the new one were recorded in *Flora of China* [18]. Leaf materials for DNA extraction were dried with silica gel. Vouchers of samples (Tab. 1) were deposited in the National Herbarium (PE), IBCAS. *Cryptocarya calcicola* H. W. Li is selected as the outgroup of *Beilschmiedia*, *Endiandra*, and *Syndiclis*. Due to lacking DNA samples and sequences in the Gene Bank, *Beilschmiedia* species outside China were not integrated into our analyses.

**Table 1:** Vouchers and accession numbers of sequences.

Species	Voucher	Locality	Accession no.			
			nrITS	*trnL-trnF*	*rpl16*	*psbA-trnH*
*Beilschmiedia appendiculata* (C.K. Allen) S.K. Lee et Y.T. Wei	*B. Liu 1504*	Guangzhou, Guangdong, China. Cult.	KC958643	KC958611	KC958675	KC958707
*B. fordii* Dunn	*B. Liu 1080*	Shangsi, Guangxi, China	KC958625	KC958593	KC958657	KC958689
*B. delicata* S.K. Lee et Y.T. Wei	*B. Liu 1451*	Gulin, Sichuan, China	KC958642	KC958610	KC958674	KC958706
*B. delicata* S.K. Lee et Y.T. Wei	*F. Q. Liu s. n.*	Enshi, Hubei, China	KC958641	KC958609	KC958673	KC958705
*B. glauca* var. *glaucoides* H.W. Li	*B. Liu 1323*	Malipo, Yunnan, China	KC958638	KC958606	KC958670	KC958702
*B. kweichowensis* Cheng	*B. Liu 1419*	Libo, Guizhou, China	KC958631	KC958599	KC958663	KC958695
*B. laevis* C.K. Allen	*B. Liu 1270*	Guangzhou, Guangdong, China. Cult.	KC958626	KC958594	KC958658	KC958690
*B. linocieroides* H.W. Li	*B. Liu 1479*	Jinping, Yunnan, China	KC958627	KC958595	KC958659	KC958691
*B. percoriacea* C.K. Allen	*B. Liu 1254*	Xishuangbanna, Yunnan, China. Cult.	KC958628	KC958596	KC958660	KC958692
*B. purpurascens* H.W. Li	*B. Liu 1321*	Malipo, Yunnan, China	KC958629	KC958597	KC958661	KC958693
*B. purpurascens* H.W. Li	*B. Liu 1443*	Malipo, Yunnan, China	KC958630	KC958598	KC958662	KC958694
*B. robusta* C.K. Allen	*B. Liu 1466*	Maguan, Yunnan, China	KC958632	KC958600	KC958664	KC958696
*B. robusta* C.K. Allen	*B. Liu 1481*	Jinping, Yunnan, China	KC958633	KC958601	KC958665	KC958697
*B. robusta* C.K. Allen	*B. Liu 1428*	Malipo, Yunnan, China	KC958634	KC958602	KC958666	KC958698
*B. rufohirtella* H.W. Li	*B. Liu 1302*	Malipo, Yunnan, China	KC958635	KC958603	KC958667	KC958699
*B. rufohirtella* H.W. Li	*B. Liu 1493*	Xichou, Yunnan, China	KC958636	KC958604	KC958668	KC958700
*B. rufohirtella* H.W. Li	*B. Liu 1430*	Malipo, Yunnan, China	KC958637	KC958605	KC958669	KC958701
*B. turbinata* Bing Liu et Y. Yang	*B. Liu 1442*	Malipo, Yunnan, China	KC958639	KC958607	KC958671	KC958703
*B. turbinata* Bing Liu et Y. Yang	*B. Liu 1185*	Malipo, Yunnan, China	KC958640	KC958608	KC958672	KC958704
*B. yunnanensis* Hu	*B. Liu 1473*	Maguan, Yunnan, China	KC958644	KC958612	KC958676	KC958708
*B. yunnanensis* Hu	*B. Liu 1484*	Jinping, Yunnan, China	KC958645	KC958613	KC958677	KC958709
*B. yunnanensis* Hu	*B. Liu 1439*	Malipo, Yunnan, China	KC958646	KC958614	KC958678	KC958710
*B. yunnanensis* Hu	*B. Liu 1474*	Maguan, Yunnan, China	KC958647	KC958615	KC958679	KC958711
*Cryptocarya calcicola* H.W. Li	*B. Liu 1457*	Malipo, Yunnan, China	KC958656	KC958624	KC958688	KC958720
*Endiandra coriacea* Merr.	*J. F. Ye s. n.*	Taizhong, Taiwan, China. Cult.	KC958655	KC958623	KC958687	KC958719
*E. dolichocarpa* S.K. Lee et Y.T. Wei	*B. Liu 1381*	Jinping, Yunnan, China	KC958653	KC958621	KC958685	KC958717
*E. dolichocarpa* S.K. Lee et Y.T. Wei	*B. Liu 1480*	Jinping, Yunnan, China	KC958654	KC958622	KC958686	KC958718
*Syndiclis anlungensis* H.W. Li	*B. Liu 1452*	Anlong, Guizhou, China	KC958648	KC958616	KC958680	KC958712
*S. marlipoensis* H.W. Li	*B. Liu 1282*	Malipo, Yunnan, China	KC958649	KC958617	KC958681	KC958713
*S. marlipoensis* H.W. Li	*B. Liu 1420*	Malipo, Yunnan, China	KC958650	KC958618	KC958682	KC958714
*Syndiclis* sp.	*B. Liu 1472*	Maguan, Yunnan, China	KC958651	KC958619	KC958683	KC958715
*Syndiclis* sp.	*B. Liu 1486*	Pingbian, Yunnan, China	KC958652	KC958620	KC958684	KC958716

Total DNA was extracted from silica-gel-dried leaves using Tiangen® extraction kits. Polymerase chain reaction amplification was accomplished using Prime Taq DNA Polymerase with annealing temperatures of 50°C. The ITS region was amplified with primer pair ITS4, ITS5 [36] and C26A, 18S [37]. The chloroplast *trnL-trnF* region was amplified using primers designed by Taberlet et al. [38]. For some special species, the sequences of *Beilschmiedia* and related genera were obtained by our own designed primers, and the suitable annealing temperature is 50°C. The *rpl16* intron and *psbA-trnH* spacer were amplified using primers of Sang et al. [39] and Kelchner et al. [40], respectively. All the primers used in this study are listed in Tab. 2.

**Table 2:** Primers used for polymerase chain reaction amplification and sequencing.

Primer	Sequence 5′-3′	Reference
ITS 4	TCCTCCGCTTATTGATATGC	White et al., 1990
ITS 5	GGAAGTAAAAGTCGTAACAAGG	White et al., 1990
ITS C26A	GTTTCTTTTCCTCCGCT	Wen et al., 1996
ITS 18S	AGGAGAAGTCGTAACAAG	Wen et al., 1996
*trnL-trnF* f	ATTTGAACTGGTGACACGAG	Taberlet et al., 1991
*trnL-trnF* c	CGAAATCGGTAGAGGCTACG	Taberlet et al., 1991
*trnL-trnF* FC	CTTAAACTCAGCGGGTGGTCC	this study
*trnL-trnF* RA	TAACAAGGTTTCCGTAGGTGAAC	this study
*rpl16* f71	GCTATGCTTAGTGTGTGACTCGTTG	Kelchner, 1997
*rpl16* r1516	CCCTTCATTCTTCCTCTATGTTG	Kelchner, 1997
*psb*A 3F	GTTATGCATGAACGTAATGCTC	Sang et al., 1997
*trn*H 3R	CGCGCATGGTGGATTCACAATCC	Sang et al., 1997

Sequences were manually aligned using the Sequence Alignment Editor (Se-Al Carbon) v2.0 [41]. Bayesian inference (BI) and Maximum parsimony (MP) analyses based on the combined datasets of nuclear ribosomal ITS and chloroplast *trnL-trnF*, *rpl16*, *psbA-trnH* were carried out in MrBayes 3.1.2 [42] and PAUP 4.0 [43]. The poly-T and poly-A regions were removed from the sequences during analyses.

### Nomenclature

The electronic version of this article in Portable Document Format (PDF) in a work with an ISSN or ISBN will represent a published work according to the International Code of Nomenclature for algae, fungi, and plants, and hence the new names contained in the electronic publication of a PLOS ONE article are effectively published under that Code from the electronic edition alone, so there is no longer any need to provide printed copies.

In addition, new names contained in this work have been submitted to IPNI, from where they will be made available to the Global Names Index. The IPNI LSIDs can be resolved and the associated information viewed through any standard web browser by appending the LSID contained in this publication to the prefix http://ipni.org/. The online version of this work is archived and available from the following digital repositories: PubMed Central, LOCKSS.

## Results

### Description of the New Species

#### Beilschmiedia turbinate

**Figure 1:**
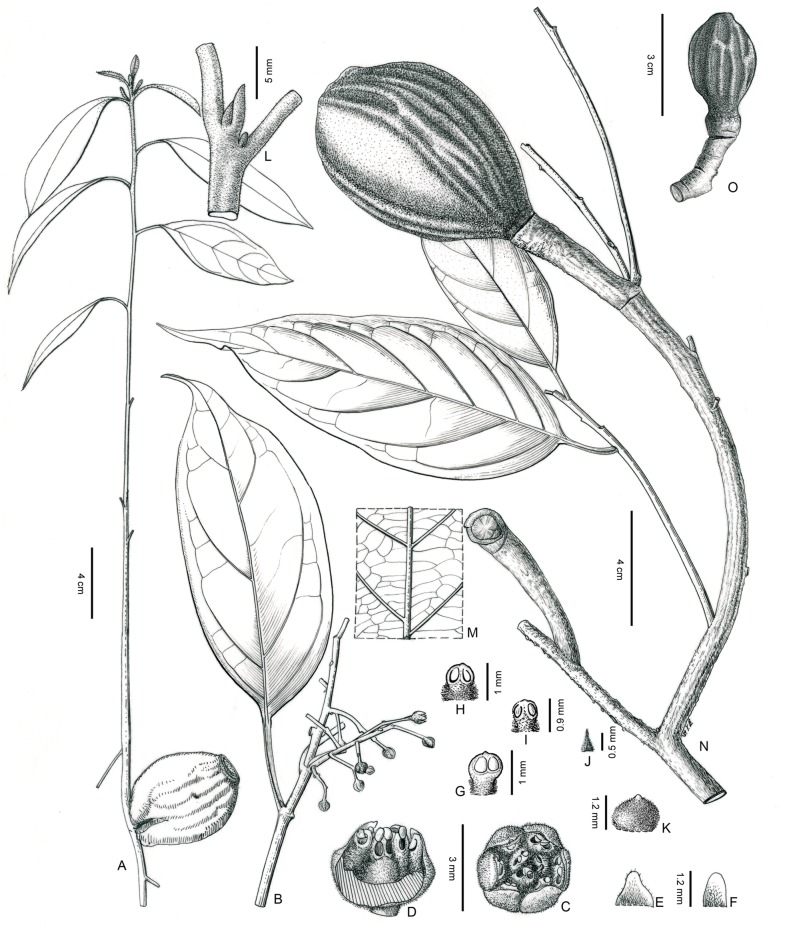
Illustrations of *Beilschmiedia turbinata* Bing Liu & Y. Yang showing morphological details. A, seedling; B, Flowering branch; C, flower; D, flower (front part removed); E, the first whorl perianth lobe; F, the second whorl perianth lobe; G. the first whorl stamen (adaxial view); H, the second whorl stamen (adaxial view); I, the third whorl stamen (abaxial view); J, the fourth whorl staminode (abaxial view); K, pistil showing the pubescent ovary; L, terminal bud; M, venation pattern (abaxial view); N, fruiting branch in the second year; O, a young fruit in the first year (Drawn by Y. B. Sun, A from *Bing Liu 1184*, B–M from *Bing Liu 1442*, N–O from *Bing Liu 1185, PE*).

**Figure 2:**
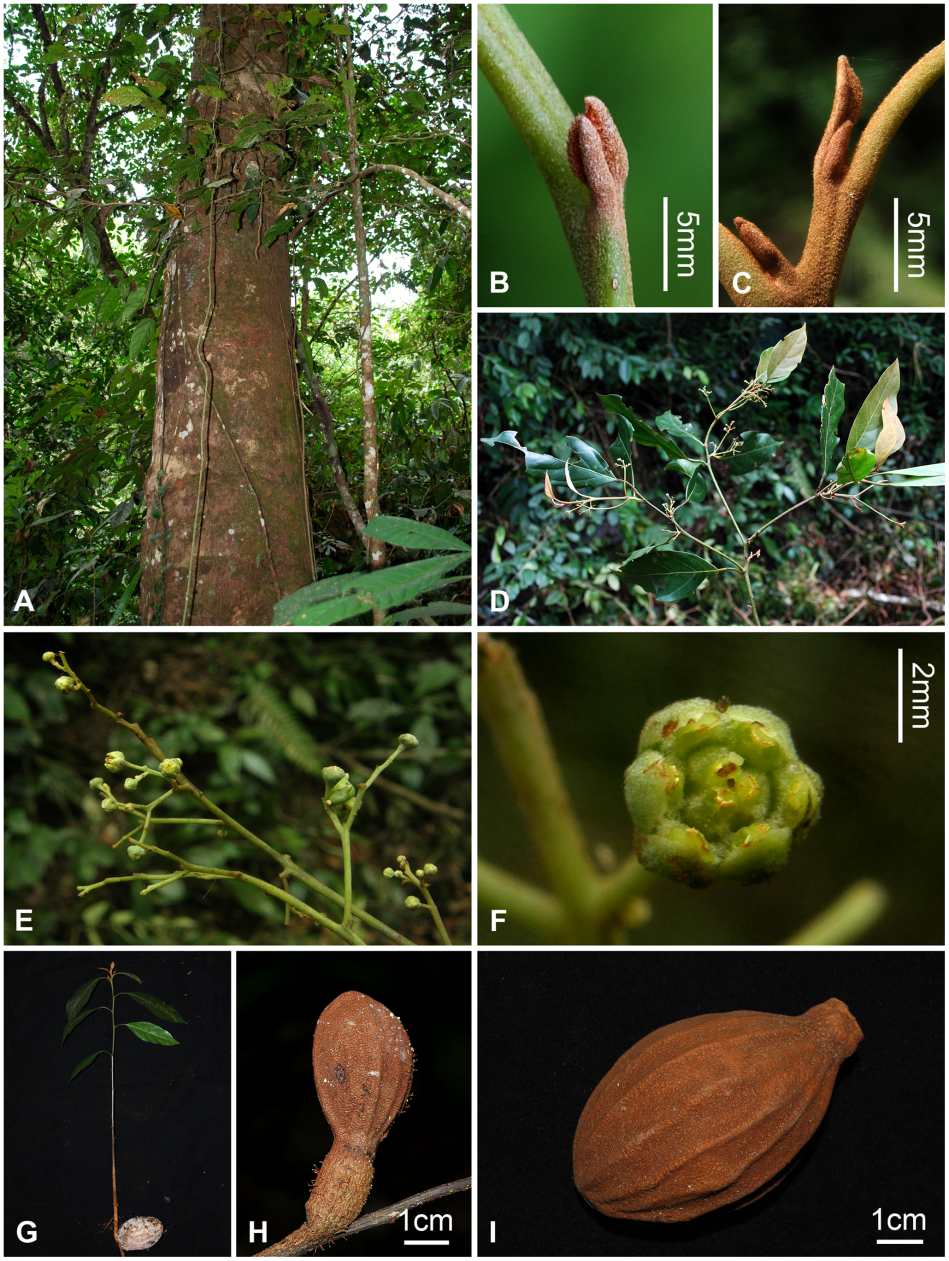
Morphology of *Beilschmiedia turbinata* Bing Liu et Y. Yang. A, trunk (showing brownish gray bark); B–C, the pubescent terminal bud; D, flowering branch; E, inflorescence; F, flower (showing the trimerosity and the pubescent tepals, the included 2-celled stamens with the outer two whorls introrse and adnate to tepals but the third whorl getting close and adnate to the pistil); G, seedling (showing the alternative leaves on the new sprout); H, a young fruit in the first year bearing a conspicuously thickened pedicel; I, a large, ferruginous-furfuraceous turbinate, mature fruit.

Bing Liu & Y. Yang sp. nov. Figs. 1, 2 77128395-1.

#### Type

China. Yunnan Province: Malipo County, Tianbao, Sumawan, Long. 104.84 E, Lat. 22.98 N, 24 Sept 2011, flowering, Bing Liu 1442 (holotype, PE).

#### Diagnosis

Haec species nova ramulis juvenilibus, gemmis terminalibus foliisque juvenilibus dense fulvo-tomentosis, glandulis staminium ordinis III absentibus, fructibus turbinatis et ferrugineo-furfuraceis, valde magnis, ad 7.2 cm longis, 4.6 cm in diametro inter alia distinguenda.

#### Description

Trees, up to 25 m tall, to 1 m in DBH (diameter at breast height). Bark brownish gray. Branchlets 1.6–2 mm in diam., grayish brown, lenticellate, green, ferruginous-brown pubescent when young and blackish brown when dry. Terminal buds small type, 4–5 mm long, densely ferruginous-brown tomentose. Leaves alternate or subopposite; leaf blade broadly lanceolate, elliptic to elliptic-lanceolate, 7–12(−16)×2.2–4.2(−6) cm, base broadly cuneate to obtuse, often oblique, apex caudate-acuminate, midrib elevated on both surfaces, lateral veins 4–6(−7) pairs, immersed on upper surface but elevated on lower surface, minor veins slender, connected into fine type of minor venation pattern, covered with dense ferruginous pubescence on both surfaces when young, but glabrescent soon; petiole 1.4–2 cm long, densely ferruginous tomentose when young. Inflorescences paniculate; panicles terminal or axillary, 4.5–8×3–5.5 cm, few flowered; rachises slightly robust, sparsely ferruginous tomentose; bracts ovate, 2–3 mm long, caducous; flowers yellowish green, ca. 1.5 mm in length, 3 mm in diam.; pedicels 2–2.5 mm, sparsely tomentose; perianth lobes 6, ovate or broadly ovate, 1.2×1 mm, pubescent on both surfaces. Fertile stamens nine in three whorls, eglandular, subequal, ca. 1 mm long, filaments short but present and densely pubescent; anthers 2-celled, cells of the first and second whorls of stamens introrse, and those of the third whorl extrorse, the tip of anthers glabrous; staminodes of fourth series long-triangular and pubescent, ca. 0.6 mm long; pistils pyriform, pubescent, style short, ca. 0.3 mm. Each infrutescence usually bearing one single mature fruit; fruits maturing in two years. Young fruits of the first year ovoid, 2.5–3×1.5–1.8 cm. Mature fruits large and turbinate, 5.4–7.2×3.8–4.6 cm, apex obtuse, base attenuate, densely ferruginous-furfuraceous, having 16–20 irregular longitudinal ridges; fruiting pedicles conspicuously thickened and robust, 1.3–1.8 cm in diam. Young seedlings up to 40 cm in height, leaves alternate on the new sprout, the cotyledons retained for one or two years.

#### Distribution

**Figure 3:**
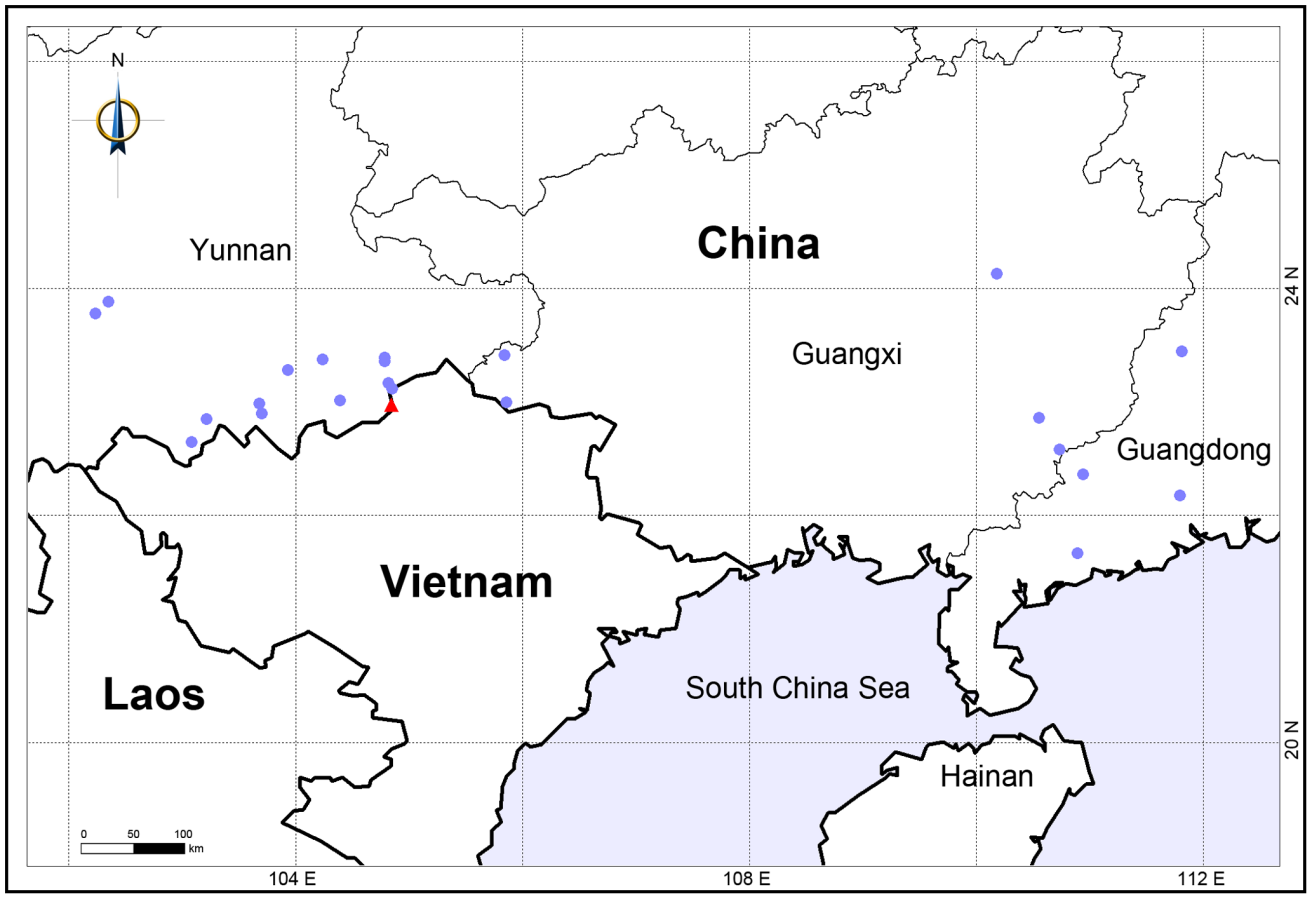
Geographical occurrences of *Beilschmiedia turbinata* Bing Liu et Y. Yong and *B. yunnanensis* Hu. Triangles represent *B. turbinata*, and circles refer to *B. yunnanensis.*

This species was only found in the border of China (Malipo County of Yunnan Province) and Vietnam (Vi Xuyen County of Ha Giang Province) (Fig. 3).

#### Habit & Ecology

The species inhabits limestone soils of primary forests on hills with altitudinal ranges from 1000 to 1200 m. Associated tree species include *Beilschmiedia purpurascens* H.W. Li, *B. delicata* S.K. Lee et Y. T. Wei, *Cinnamomum saxatile* H.W. Li, *Actinodaphne obovata* (Nees) Blume, *Litsea yunnanensis* Y.C. Yang et P.H. Huang, *Lindera gracilipes* H.W. Li, *Cryptocarya* sp., *Caryodaphnopsis* sp., *Cyclobalanopsis* spp., *Lithocarpus* sp., *Garcinia* sp., *Syzygium austroyunnanense* H.T. Chang et R.H. Miao, *Aglaia testicularis* C.Y. Wu, *Amoora* sp., *Pittosporum* sp., and *Ostodes katharinae* Pax. It blooms from September to October, and fruiting season is from October to November; it takes two years for fruits to get mature.

#### Conservation

There is only one population with ca. 30 adult trees across the boundary of China and Vietnam. These mature individual trees are dispersed in the evergreen broad-leaved forest of ca. 20 km^2^ (EOO). The trees were cut down for construction leading to the population decline. Moreover, increasing human economic activities, e.g. the surrounding reclamation of farmlands, construction of border roads, and rubber plantations, are resulting in the fragmentation of the population, and threatening the existence of the new species and its living habitat. Our field observation found dozens of seedlings nearby the parent trees, but no young sapling exists. Alien species (e.g. *Chromolaena odorata* (L.) R. M. King et H. Rob. and *Crassocephalum rubens* (Jacq.) S. Moore) are also found nearby. Consequently, we here tentatively consider the new species to be “Critically Endangered” (CR Blab (v)+D), according to IUCN red list category and criterion [34].

#### Etymology

The specific epithet “*turbinate*” of this new species is from its turbinate shape of fruits.

#### Additional specimens examined

China. Yunnan Province: Malipo County, Tianbao, Sumawan, Long. 104.84 E, Lat. 22.98 N, 15 Nov 2010, fruits, Bing Liu 1185 (PE); Vietnam. Ha Giang Province: Vi Xuyen County, Thanh Thuy, Long. 104.86 E, Lat. 22.98 N, 15 Nov 2010, seedling, Bing Liu 1184 (PE).

### Systematics

**Figure 4:**
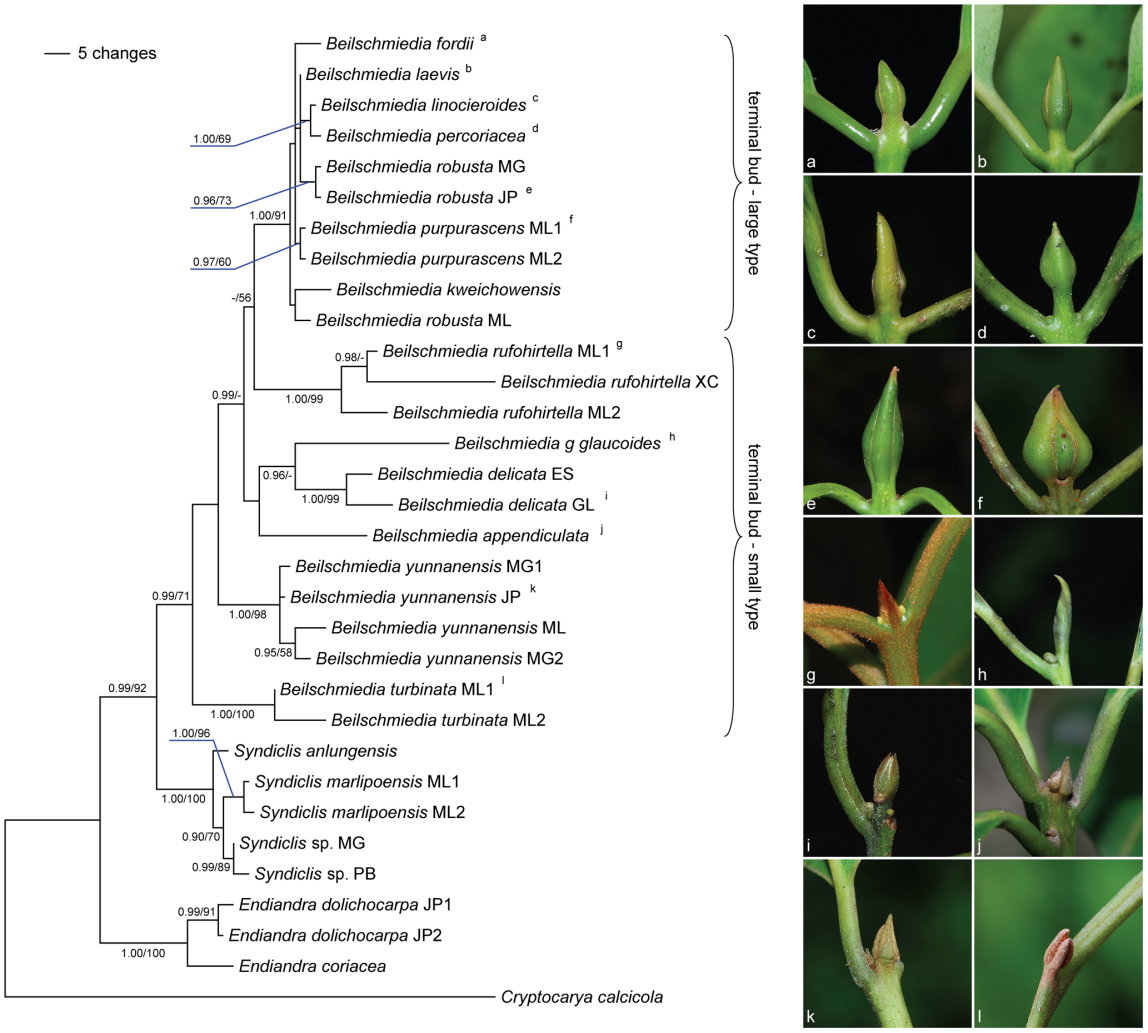
Bayesian consensus of 18001 trees based on combined sequence data of nrITS and *trnL-trnF*, *rpl16*, *psbA-trnH* analysis. Bayesian posterior probability values greater than 0.9 (left) and bootstrap support value greater than 50 (right) are shown on the branches. The different types of terminal buds are illustrated on the right.

Partition homogeneity test between nrITS and chloroplast sequences was carried out using PAUP and the P value is 0.07. Therefore, we combined them into one dataset, representing 2618 characters, including 163 characters that were parsimony-uninformative and 135 characters that were parsimony-informative. Analyses of nrITS, *trnL-trnF*, *rpl16*, *psbA-trnH* and combined regions were carried out using MP (heuristic search) and the results are shown in Tab. 3, and the Bayesian consensus tree based on combined sequence data is shown in Fig. 4.

**Table 3:** Summary of parsimony analyses and data properties for nuclear ribosomal internal transcribed spacer (nrITS), chloroplast *trnL-trnF*, *rpl16*, *psbA-trnH*, and combined regions.

DNA region	ITS	*trnL-trnF*	*rpl16*	*psbA-trnH*	combined
**Length of sequences [bp]**	600–642	597–614	775–803	463–502	2496–2547
**Length of alingment [bp]**	668	626	774	508	2618
**Variable sites (%)**	70 (10.48)	35 (5.59)	36 (4.65)	22 (4.34)	163 (6.22)
**Parsimony-informative sites (%)**	78 (11.68)	36 (5.75)	6 (0.78)	15 (2.95)	135 (5.16)
**Consistency index (CI)**	0.7577	0.6847	1.0000	0.9268	0.7466
**Retention index (RI)**	0.8476	0.6196	1.0000	0.9483	0.7910
**Rescaled consistency index (RC)**	0.6423	0.4242	1.0000	0.8789	0.5906

The result indicates that *Syndiclis* and *Beilschmiedia* are reciprocally monophyletic with the monophyletic *Endiandra* as outgroup, and *Beilschmiedia turbinata* clearly belongs to *Beilschmiedia*, though the position of the species is not well resolved within the genus (Fig. 4). In addition, this new species has typical *Beilschmiedia* morphology such as the trimerous flowers, nine fertile stamens in three whorls, fruits lacking persistent perianth lobes. This suggests that the new species is a *Beilschmiedia* but markedly differs from *Syndiclis* and *Endiandra*. *Beilschmiedia turbinata* shows great similarities to *B. yunnanensis* in morphology, but it is not conclusive whether *B. turbinata* and *B. yunnanensis* are basal to other *Beilschmiedia* species or not due to the low bootstrap value (<50%).

## Discussion

### Morphological Comparison

**Figure 5:**
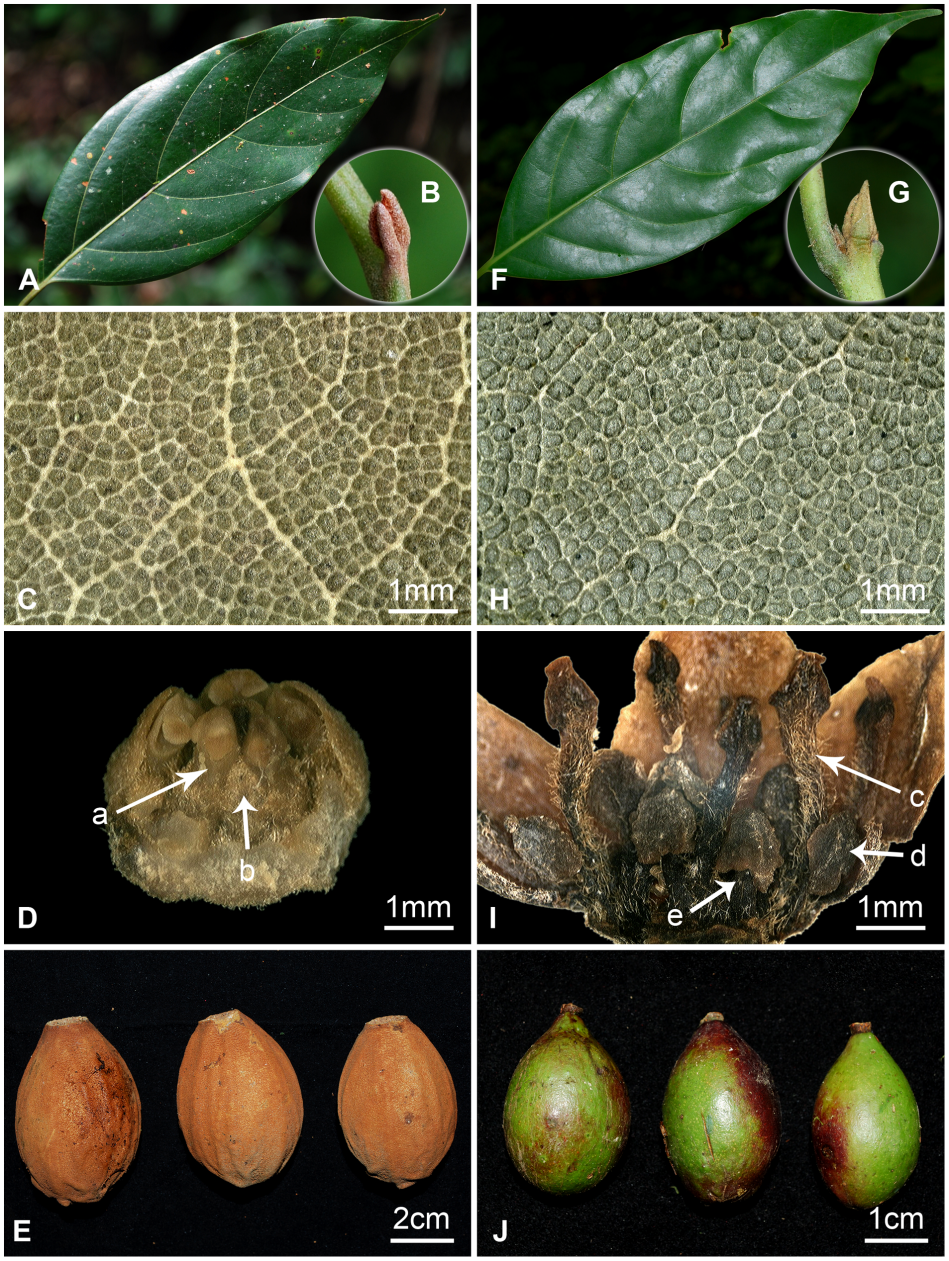
Comparison between *Beilschmiedia turbinata* Bing Liu et Y. Yang and *B. yunnanensis* Hu. A–E, *B. turbinata* Bing Liu & Y. Yang. A, leaf blade; B, the small type terminal bud bearing ferruginous-brown pubescence; C, leaf upper surface showing fine vein reticulation; D, flower (front part removed showing: a, the third whorl subsessile fertile stamen; b, the fourth whorl staminode ); E, the large, ferruginous-furfuraceous turbinate fruits. F–J, *B. yunnanensis* Hu. F, leaf blade; G, the small type terminal bud bearing brownish pubescence; H, leaf upper surface showing fine vein reticulation; I, flower (front part removed showing: c, the third whorl stalked and glandular fertile stamen; d, the sagittate gland at the base of the third whorl stamen; e, the fourth whorl staminode); J, the smaller ellipsoid fruits.

*Beilschmiedia turbinata* possesses a unique set of morphological characters, e.g. the small type of terminal buds densely pubescent, elliptic to lanceolate elliptic leaves having fine veinlet reticulations, the trimerous flowers bearing nine eglandular fertile stamens in three whorls, and the large ferruginous-furfuraceous turbinate fruits. This new species is similar to *B. yunnanensis* in the small type and ferruginous-brown tomentose terminal buds, alternate or subopposite elliptic to oblong-lanceolate leaves, the elevated midrib, and the fine veinlet reticulation (Fig. 5), but differs from the latter by the smaller flowers (3 mm in diam. in the new species vs. up to 8 mm in *B. yunnanensis*), the eglandular stamens of the third whorl (vs. each of the three fertile stamens of the third whorl bearing two sagittate glands in *B. yunnanensis*), and the ovoid to turbinate, large, 5.4–7.2×3.8–4.6 cm, and densely ferruginous-furfuraceous (vs. ellipsoid to globose or subglobose, smaller, 2–4×1.5–2.7 cm in *B. yunnanensis*) (Fig. 5).

*Beilschmiedia turbinata* also shares similar characters with *B. dictyoneura* Kosterm. from Borneo in having eglandular stamens [17], but they are easily distinguished: *B. dictyoneura* bears glabrous twigs and leaves, larger terminal buds, and smaller fruits, 1.5 cm×1 cm [17].

A few species from Southeast Asia also bear large fruits, e.g. *Beilschmiedia macrocarpa* A. Chev. ex H. Liou from S Vietnam, with the ovoid fruits up to 6.2 cm [14]; it differs from *B. turbinata* by its large terminal buds (vs. small terminal buds) and coarse veinlet reticulation (vs. fine veinlet reticulation). *Beilschmiedia gigantocarpa* Kosterm. from Sulawesi, *B. glauciphylla* Kosterm., *B. gynotrochioides* Kosterm. and *B. kinabaluensis* Kosterm. from Borneo, all bear fruits larger than 5 cm in diam. [17], [44]. *Beilschmiedia turbinata* can be easily distinguished from them by the turbinate shape of fruits vs. spindle-shaped shape of fruits in *B. glauciphylla* and *B. gynotrochioides*, globose to subglobose shape of fruits in *B. gigantocarpa* and *B. kinabaluensis*.

### Phylogenetic and Morphological Implications

*Beilschmiedia* is mixed with allied genera (e.g. *Potameia* and *Endiandra*) according to previous studies [24], [29]. Sampling in these studies is poor, there are only six species of *Beilschmiedia*, two of *Potameia*, and one of *Endiandra* in Chanderbali et al. [24], seven species of *Beilschmiedia*, one of *Potameia*, and three of *Endiandra* in Rohwer & Rudolph [29], and these samplings are common in lacking Asiatic species. We sampled nineteen Asiatic species of the group (mostly from Yunnan) with the highest sampling density of the *Beilschmiedia* group so far. Our new phylogenetic analysis suggested that the new species belongs to the genus *Beilschmiedia*.

Morphology of the terminal buds is useful in classification of the genus *Beilschmiedia* [16]–[18], [45]–[46]. Hooker [44] even divided *Beilschmiedia* into two sections mainly by the type of terminal buds: Sect. I, leaves opposite or alternate, terminal buds very small, pubesent or tomentose, not enclosed in coriaceous scales; Sect. II, leaves usually opposite, terminal buds enclosed in large glabrous coriaceous scales. According to the phylogenetic tree of this study, species bearing large type of terminal buds do constitute a well-supported clade (Fig. 4), and are considered to be derived from the species of small type. *Beilschmiedia yunnanensis* and *B. turbinata*, occupying the basal position of this genus, both have the small type terminal bud. Worldwide sampling of the *Beilschmiedia* group is necessary to test and verify whether this character is useful for subgeneric classification or not.

### Conservation Significance

Conservation status of *B. turbinata* warrants our attention. The new species is an endangered species and listed as CR, which is attributed to population decline, habitat loss, and alien species invasions because of increasing human activities in the area. Large trees of the species were cut down for construction. Expansion of farmlands and rubber plantations results in deforestation, and of course, habitat loss and fragmentation of population of the species. Road building causes fragmentation of population of the new species. Alien species invasions had potential impacts on both seed germination and growth of seedlings, and young seedlings might be difficult to grow up because of the lean soils and pressure of competition from both native and alien species. These factors are also major threats of regional biodiversity in Tropical Asia [5], [9].

Yunnan Province is located in the SW China, harbors more than half the total species in China, and is the province with the highest floristic richness [47]. Within Yunnan Province, the southeastern and southern regions are the most important and abundant center of plant diversity. More than 7000 species of flowering plants are native to the Southeast Yunnan alone [7], and over 4000 species are reported from southern Yunnan [48]. However, Yunnan remains an active area for plant taxonomic studies, there are 55 publications every year related to “new species”+“Yunnan”+“plant” during 2000 and 2012 according to ISI website searching. The floristic inventory of Yunnan is far from being completed, and description of new species may not catch up with the extinction of local plants due to over exploitation, habitat loss, and invasion of alien species.

The local governments have made great efforts to develop cash crops in the tropical areas, especially in southern Yunnan and lowlands of southeast Yunnan. Plantations occupy a large area previously occupied by old-growth primary forests, leading to deforestation and environmental destruction much more serious than several decades ago. Wang [49] took part in the China-Russia joint expedition to southern Yunnan in 1950’s, and revisited the same area in 1990’s. According to his observation in the air and on-the-spot investigation, both appearance and composition of the forests in southern Yunnan had greatly changed within the 40 years. The canopy color of the forest was dark green with very thick forests in 1950’s, but was changed into light green with many open places in 1990’s. Many of the primary forests with abundant species were cut down and transformed into economic monoculture forests (e.g. rubber), and the local biodiversity in south Yunnan was precipitously reduced. Transformation of forest structure has negative effects not only on plant species, but also on insects [3]. The rubber plantations are less than 5 kilometers away from the forest where our new species was collected. Local villagers are unaware of protection, but still utilize the timbers by logging the large trees and substituting the forests with economic trees. The rapid economic development mode at the price of destruction of native vegetation was called into questions and even caused a lot of criticism [2], [49]–[50]. Vegetation shifts caused by human disturbance have led to changes of floristic components and severe species loss [4]. *Beilschmiedia turbinata*, probably once a more widespread species, but became a dying species in SE Yunnan, sounds the alarm again that conservation of the primary forests is urgent. *Nyssa yunnanensis* W.Q. Yin ex H.N. Qin & Phengklai of Nyssaceae also experienced a similar process [51]. As a result, conservation of primary vegetation and endangered species of this biodiversity hot spot is quite worrying, conservation action is necessary. This finding furthers that botanical investigations are urgently needed before the primary forests are cut down and replaced by economic crops, e.g. rubber, eucalyptus, and banana.
